# Totally implantable venous access ports and associated complications in sub-Saharan Africa: a single-centre retrospective analysis

**DOI:** 10.3332/ecancer.2022.1389

**Published:** 2022-05-12

**Authors:** Kahmil Salawu, Oreoluwa Arowojolu, Oluwasegun Afolaranmi, Mutiu Jimoh, Chukwumere Nworgu, Bode Falase

**Affiliations:** 1 Lakeshore Cancer Center, Lagos, 101241, Nigeria; 2 Yale School of Public Health, New Haven, CT 06510, USA; 3 Nuffield Department of Surgical Sciences, University of Oxford, OX3 7DQ, UK; 4 University College Hospital, Ibadan, 200212, Nigeria; 5 Roswell Park Comprehensive Cancer Centre, Buffalo, NY 14203, USA; 6 Lagos State University Teaching Hospital, Lagos, 23401, Nigeria; † These authors are co-first authors.

**Keywords:** totally implantable venous access port (TIVAP), chemotherapy, cancer, complications, sub-Saharan Africa

## Abstract

**Purpose:**

Although totally implantable venous access ports (TIVAPs) have been safe and valuable in the management of cancer and other chronically ill patients who require long-term intermittent venous access, a few complications have been reported with their use. Data on the use of TIVAPs in sub-Saharan Africa and other low- and middle-income regions is limited. In this study, we determine the complications that arise from TIVAP use at a cancer centre in Nigeria.

**Patients and Methods:**

Between 4 January 2018 and 15 September 2020, 100 patients received TIVAPs at our centre, primarily for the administration of chemotherapy for the treatment of solid tumours. Data were retrospectively extracted from the institutional electronic medical records and descriptive analysis of patient and disease characteristics, port-specific data and data on complications and outcomes was conducted

**Results:**

The 100 patients who were implanted with TIVAPs at our cancer centre had their devices *in situ* for a total of 27,183 days, with a mean duration of use of 272 catheter-days (SD: 267 days; range: 2–952). TIVAP-related complications were identified in 13 patients (13%), i.e., an incidence of 0.478 complications/1,000 catheter-days. The mean time to onset of complications was 61 days (SD: 105 days; median: 23 days; range: 0–389). The complications observed include port-site bleeding, pocket infection, cutaneous site infection, arterial puncture, wound dehiscence, difficult access (due to port malpositioning and port site fibrosis) and others. No deaths, pneumothorax, haemothorax, catheter occlusions, or catheter-associated venous thromboses were recorded.

**Conclusion:**

Our study shows that TIVAPs can be used successfully in our environment and presents a case for more widespread use to improve both the patient experience and the ability of healthcare providers to deliver optimal treatment.

## Introduction

Totally implantable venous access ports (TIVAPs), otherwise known simply as subcutaneous venous ports, are small medical devices used in patients who require long-term intermittent venous access for prolonged therapies. They have been of tremendous use in the management of cancer patients for long-term chemotherapy infusions, hydration, parenteral nutrition and serial blood withdrawals, making treatment more comfortable and convenient [1, 2]. The contemporary port system is composed of an indwelling central catheter inserted into the subclavian vein or the internal jugular vein and attached to a reservoir (a port chamber placed in a subcutaneous pocket) which can be accessed percutaneously by a special needle under sterile conditions. Thus, the device is invisible and patients are able to perform normal activities like showering and swimming when it is not in use [3].

Although TIVAPs have improved the lives of patients who require their use, there have been some complications reported with their use. Overall, complications associated with the use of TIVAPs appear to occur in up to 31% of the cases [4]. Common complications that have been reported in previous studies include malposition of the catheter/reservoir, skin perforation, wound dehiscence, pneumothorax or haemothorax, which generally occur in the early post-insertion period, as well as late complications, including drug extravasation, mechanical malfunction, catheter migration, catheter fracture, catheter disconnection, vein thrombosis, thromboses of the reservoir/catheter, skin infection, skin necrosis and sepsis [1, 5].

Unfortunately, TIVAPs do not appear to be widely used in countries in sub-Saharan Africa and other low- and middle-income regions. Likely challenges facing widespread adoption of implantable vascular port systems in these environments include high cost, port device unavailability and lack of required surgical skill for implantation. Consequently, there is a paucity of literature regarding TIVAP use and associated complications emanating from these regions. The limited data could further inhibit widespread use of these devices in the region, as providers might not be confident in their use. In this study, we therefore present the experience with TIVAP usage and associated complications at a cancer centre in Nigeria.

## Patients and methods

One hundred patients received TIVAPs at our specialist oncology centre in Lagos, Nigeria, between 4 January 2018 and 15 September 2020, primarily for the administration of chemotherapy, including induction, consolidation, maintenance, adjuvant/neoadjuvant and palliative chemotherapy. Patients received TIVAPs on the basis of difficulty in accessing peripheral venous access. Patient and disease characteristics (Table 1), port-specific data (Table 2) and data on complications and outcomes (Table 3) were retrospectively extracted from institutional electronic medical records for analysis.

Every patient received a single type of TIVAP made up of plastic and silicone, connected to a flexible 8F polyurethane catheter (Dignity→; Medcomp; Harleysville, PA, USA). This was the device of choice as it was the only device available with adequate expertise for its insertion. The port systems were implanted under local anaesthesia in the operating room under sterile conditions using the Seldinger technique, often via the right internal jugular vein; however, in certain conditions which could impact placement or therapy (e.g., a right breast mass), the ports were placed on the left. Port placements were carried out with ultrasound guidance (real-time ultrasound using out-of-plane technique) without fluoroscopic guidance, and forward flow and back flow were always tested via the pocket after insertion. Thereafter, a confirmatory chest x-ray was always performed in order to promptly detect malpositioning, kinking or other complications, such as pneumothorax.

Based on institutional protocol, ports were only accessed by either a doctor or an experienced nursing staff who ensured ports were flushed with normal saline and locked with heparin after every session of infusion or blood withdrawal. In instances of prolonged disuse of the port devices, they were checked for patency, flushed with saline and locked with heparin routinely every 6 weeks.

Port device duration of use, in days, was calculated as time from implantation to either removal after concluding therapy, death of patient, loss to follow-up/transfer of care or 15 September 2020, in cases of ongoing use. Time to complication (TTC) indicates time from implantation to first day of onset of complication.

For the purpose of this study, complications were categorised into immediate (within 24 hours), early (≤30 days) or late (>30 days). Major complications were defined as complications requiring either surgery or prolonged medical therapy with hospital stay >24 hours, or resulting in death. Port-site pain was reported as a complication only if it occurred for >48 hours after implantation. Cutaneous site infection was defined as induration, erythema, tenderness and exudate around the port, with or without clinical/laboratory evidence of systemic sepsis. Port pocket infection was defined as induration, erythema and tenderness around the port with purulent material aspirated from the port pocket. Patients, with bleeding from port site, were evaluated for thrombocytopenia. Catheter-related venous thrombosis was only assessed clinically due to the unavailability of regular ultrasonographic or phlebographic monitoring.

### Data analysis

Data was analysed using IBM Statistical Package for Social Sciences (SPSS) version 23.0. Descriptive statistics, including frequencies, means and standard deviations, were computed as appropriate.

## Results

During the period studied, 100 patients who were implanted with TIVAPs at our cancer centre had their devices *in situ* for a total of 27,183 catheter-days, with the mean duration of use being 272 catheter-days (SD: 267 days; range: 2–952). Of these, 29 patients had discontinued their port devices due to conclusion of therapy or death, 4 patients were lost to follow-up or transferred care, while 67 patients still had ongoing use as at the time of data collection. Table 2 summarises device port-specific data. Asides chemotherapy for their primary solid malignancies, all patients received other intravenous therapies, such as antibiotics, fluids, antiemetics, analgesics and transfusions through their TIVAPs. In addition, the ports were used for serial blood withdrawals, with no problems encountered.

TIVAP-related complications were identified in 13 patients (13%) in this study, corresponding to an incidence of 0.478 complications/1,000 catheter-days. The TTC was 61 catheter-days (SD: 105 days; median: 23 days; range: 0–389). There was one immediate complication, seven early complications and five late complications. No major complications or deaths were observed. No haemothorax or pneumothorax was recorded in this series. One patient in this series had accidental arterial puncture, which did not result in any major adverse outcome. All three patients who complained of port-site pain >48 hours after implantation experienced relief with analgesics. An overview of the complications observed and their characteristics is presented in Table 3.

One of the three patients who experienced bleeding from the port site was managed with wound dressing only, while the other two had laboratory evidence of thrombocytopenia and required transfusion. Difficulty in accessing the port was recorded in two patients. This was due to extensive fibrosis and scarring at the port placement site due to a previous port implantation in one case and the other was noted to be a result of port malpositioning. The former required re-implantation to a different site. In the case of the latter, which occurred 21 days after insertion, the port was found turned upside down during surgical exploration and its function was restored following readjustment with no further complications. Notably, there was no incidence of drug extravasation, catheter rupture and embolisation, catheter occlusion or catheter-associated venous thrombosis reported in this series. Cutaneous site infection was observed in one patient and managed with appropriate systemic antibiotics and wound dressing. Port-pocket infection was recorded in two patients in this study. In one case, *Staphylococcus aureus* was cultured from the aspirate and the patient was managed successfully with systemic antibiotics only. However, in the second patient, the aspirate yielded growth of *Klebsiella pneumoniae* and *Pseudomonas aeruginosa,* and port removal and systemic antibiotic therapy were ultimately required to achieve a cure prior to re-implantation.

In this study, we found no statistically significant association between the development of complications and any of age group, body mass index (BMI), Eastern Cooperative Oncology Group (ECOG) performance status, disease stage, co-morbidities (hypertension or diabetes mellitus) or site of implantation.

## Discussion

TIVAPs have been in use for over three decades and provide comfort and convenience for patients and healthcare providers. Also, their low rates of extravasation make serial administration of toxic medications like chemotherapy tolerable [6]. However, despite their benefits, there is limited information about their use in sub-Saharan Africa and other low- and middle-income regions. Here, we report an overall port-related complication rate of 13%, and complication incidence of 0.478 complications/1,000 catheter-days. This appears moderately lower than those reported in recent studies [7–9]. A retrospective review of long-term complications in 81 patients carried out in another developing country observed a complication rate of 31% (1.253 complications/1,000 catheter-days), which is markedly higher than the incidence reported in our study [4].

Complications unique to the subclavian vein catheter, such as ‘catheter pinch-off’, chylothorax, and brachial plexus injury, do not occur with internal jugular vein catheters. Also, in comparison to subclavian catheters, internal jugular vein catheters are unlikely to result in a pneumothorax [10, 11]. Port-site pain after TIVAP placement should subside after 24–48 hours in most patients. For this reason, we report pain occurring for >48 hours after implantation as a complication in this study. We observed no case of symptomatic catheter-related venous thrombosis. However, regular ultrasonographic or phlebographic monitoring was not carried out, thus limiting the diagnosis of venous thrombosis to clinically obvious cases. Routine use of ultrasonography and fluoroscopy during catheter placement remains highly recommended. Although ‘blind’ catheter placement has been reported to have a high success rate [12], the costs of detecting and treating potentially lethal complications of wrong catheter placement far exceed the costs of ultrasonography or intracavitary ECG to confirm the position of the catheter tip.

This study supports the widely reported conclusion that TIVAPs have low infection rates, even in spite of the often-immunocompromised state of many of the patients who require their use. The infectious morbidity of TIVAPs is likely lower than that of partially implantable systems, such as the Hickman and Groshong catheters, because TIVAPs are irrigated less frequently, require no home care and are less susceptible to contamination when not accessed [12]. Port-pocket infection, usually caused by Gram-positive cocci, suggests direct inoculation or migration of organisms along the accessing needle as the primary mechanism [12]. However, other organisms, such as *K. pneumoniae* and *P. aeruginosa,* as in one instance in this study, could also be implicated, especially in patients with compromised host defence mechanisms.

Bleeding from the port site is a complication of concern in patients with thrombocytopenia, which is not infrequent in patients on myelosuppressive medications. A retrospective analysis of 1,200 consecutive TIVAP implantations found no significant differences in the rates of port-site bleeding between patients with normal platelet counts and patients with mild to severe thrombocytopenia under platelet substitution [13]. Therefore, patients with laboratory evidence of thrombocytopenia who are planned for TIVAP placement or develop port-site bleeding post-implantation should have their platelet levels optimised.

No incidence of port occlusion was reported in this study. Lowering the risk of occlusive complications can be achieved with appropriate flushing and locking procedures [14]. Saline flushing with heparin locking was routinely done at 6-week intervals when the device was not in use.

Although this study is one of only few studies on TIVAP complications in our setting, notable limitations include small number of patients and the monocentric nature. Our findings, however, provide critical insights into TIVAP use and complications and make a case for its use to deliver long-term therapy in resource-limited settings.

## Conclusion

TIVAPs provide comfortable and convenient vascular access for the long-term management of chronic diseases, especially cancer, and their use is relatively safe with very rare incidence of life-threatening complications. Moreover, with experienced hands at insertion and excellent nursing care through the duration of use, most minor to moderate complications can be prevented, or at the least, detected early and treated promptly.

Notwithstanding the good safety profile and the increasing burden of cancer and other chronic diseases requiring long treatment durations, the use of these devices is not widespread in sub-Saharan Africa and other low- and middle-income countries for reasons not distant from cost, limited availability of the device and insufficient trained personnel. Our study, as well as others, has shown that TIVAPs can be used successfully in our environment and presents a case for more widespread use to improve both patient experience and the ability of healthcare providers to deliver optimal treatment.

## Authors’ contributions

**Conception and design**: Khamil Salawu, Mutiu Jimoh, Oreoluwa Arowojolu and Oluwasegun Afolaranmi.

**Administrative support**: Mutiu Jimoh, Khamil Salawu and Chukwumere Nworgu.

**Provision of study materials or patients**: Chukwumere Nworgu, Mutiu Jimoh and Bode Falase.

**Collection and assembly of data**: Oreoluwa Arowojolu, Oluwasegun Afolaranmi and Khamil Salawu.

**Data analysis and interpretation**: Oluwasegun Afolaranmi and Oreoluwa Arowojolu.

**Manuscript writing**: Oreoluwa Arowojolu, Oluwasegun Afolaranmi, Mutiu Jimoh, Chukwumere Nworgu, Khamil Salawu and Bode Falase.

**Final approval of manuscript**: all authors.

**Accountable for all aspects of the work**: all authors.

## Conflicts of interest

The authors declare no competing interests.

## Funding

No funding was received for this study.

## Figures and Tables

**Table 1.** Patient and disease characteristics.

Variable	Mean (±SD) or Number/100
Age	53.5 (±12.7)
Sex	Male, 18; Female, 82
Overweight/Obese (BMI ≥ 25)	75
ECOG performance status ≥ 2	15
Disease stage IV	63
Comorbidities	Hypertension, 42; Diabetes, 9
**Tumour type**	**Number/100**
Breast cancer	51
Gastrointestinal cancer	18
Gynaecological cancer	16
Lung and pleural cancer	4
Genitourinary cancer	4
Head and neck cancer	3
Musculoskeletal cancer	3
Skin cancer	1

**Table 2.** Port-specific data.

Variable	Number/100
Right internal jugular vein	79
Left internal jugular vein	21
Ongoing use	67
Completed therapy	29
Lost to follow-up/transferred care	4
Re-implantation	2

**Table 3.** Immediate, early and late complications.

Complication	No. of patients	TTC (days)	Incidence per 1,000 days of port use	Actions taken
Port-site pain	3	72 (±75)^a^	0.110	Analgesics
Port-site bleeding	3	131 (±183)^a^	0.110	Wound dressing Platelet transfusion
Difficult access (mispositioning, fibrosis)	2	11.5 (±9.5)^a^	0.074	Surgical re-adjustment Port removal and re-implantation
Pocket infection	2	53.5 (±30.5)^a^	0.074	Antibiotics Port removal and re-implantation
Cutaneous site infection	1	33	0.037	Antibiotics
Wound dehiscence	1	27	0.037	Wound dressing
Arterial puncture	1	Immediate	0.037	Manual compression

TTC = Time to complication in days.

a Mean (±SD).
